# A deficiency of apoptosis inducing factor (AIF) in Harlequin mouse heart mitochondria paradoxically reduces ROS generation during ischemia-reperfusion

**DOI:** 10.3389/fphys.2014.00271

**Published:** 2014-07-22

**Authors:** Qun Chen, Karol Szczepanek, Ying Hu, Jeremy Thompson, Edward J. Lesnefsky

**Affiliations:** ^1^Division of Cardiology, Department of Internal Medicine, Pauley Heart Center, Virginia Commonwealth UniversityRichmond, VA, USA; ^2^Department of Biochemistry and Molecular Biology, Virginia Commonwealth UniversityRichmond, VA, USA; ^3^McGuire Department of Veterans Affairs Medical CenterRichmond, VA, USA

**Keywords:** reactive oxygen species, electron transport chain, apoptosis, poly(ADP-ribose)

## Abstract

**Background and Aims:** AIF (apoptosis inducing factor) is a flavin and NADH containing protein located within mitochondria required for optimal function of the respiratory chain. AIF may function as an antioxidant within mitochondria, yet when released from mitochondria it activates caspase-independent cell death. The Harlequin (Hq) mouse has a markedly reduced content of AIF, providing an experimental model to query if the main role of AIF in the exacerbation of cell death is enhanced mitochondrial generation of reactive oxygen species (ROS) or the activation of cell death programs. We asked if the ROS generation is altered in Hq heart mitochondria at baseline or following ischemia-reperfusion (IR).

**Methods:** Buffer perfused mouse hearts underwent 30 min ischemia and 30 min reperfusion. Mitochondrial function including oxidative phosphorylation and H_2_O_2_ generation was measured. Immunoblotting was used to determine the contents of AIF and PAR [poly(ADP-ribose)] in cell fractions.

**Results:** There were no differences in the release of H_2_O_2_ between wild type (WT) and Hq heart mitochondria at baseline. IR increased H_2_O_2_ generation from WT but not from Hq mitochondria compared to corresponding time controls. The complex I activity was decreased in WT but not in Hq mice following IR. The relocation of AIF from mitochondria to nucleus was increased in WT but not in Hq mice. IR activated PARP-1 only in WT mice. Cell injury was decreased in the Hq mouse heart following *in vitro* IR.

**Conclusion:** A deficiency of AIF within mitochondria does not increase ROS production during IR, indicating that AIF functions less as an antioxidant within mitochondria. The decreased cardiac injury in Hq mouse heart accompanied by less AIF translocation to the nucleus suggests that AIF relocation, rather than the AIF content within mitochondria, contributes to cardiac injury during IR.

## Introduction

Apoptosis inducing factor (AIF) is a nuclear encoded protein synthesized as a 67 kDa precursor (Sevrioukova, 2011; Natarajan and Becker, 2012). The mature form of AIF (62 kDa) is located within the mitochondrial intermembrane space following import and removal of the mitochondrial localization signal (Sevrioukova, 2011; Natarajan and Becker, 2012). AIF has a pro-survival role when it is located within mitochondria, whereas release of AIF from mitochondria into cytosol followed by nuclear import activates caspase-independent cell death (Sevrioukova, 2011; Natarajan and Becker, 2012). The presence of FAD and NAD cofactors in the mature AIF renders it a potential antioxidant within the mitochondrial intermembrane space (Klein et al., 2002; van Empel et al., 2005), although this concept has been challenged (Sevrioukova, 2011). Nonetheless, the presence of these cofactors strongly suggests that AIF responses both within mitochondria and following release are likely responsive to and modulated by the local redox environment (Sevrioukova, 2011). The lower expression of AIF in Harlequin (Hq) mice impacts metabolism and response to tissue stress, in an organ-dependent manner (Klein et al., 2002; Vahsen et al., 2004). AIF deletion is embryonic lethal (Klein et al., 2002). When activated following birth, AIF deletion in heart and skeletal muscle leads to profound dilated cardiomyopathy and muscle wasting due to a marked decrease in electron transport complex I activity concomitant with increased reactive oxygen species (ROS) production from mitochondria (Joza et al., 2005; Pospisilik et al., 2007). Attenuation of the severity of AIF deficiency through extensive backcross breeding or the use of (female) heterozygotes leads to more modest defects in mitochondrial respiration that do not exhibit increased ROS production in the baseline state, and in fact display potential resistance to exogenous disease (Pospisilik et al., 2007) suggestive of protective modulation of metabolism (Chen et al., 2007; Oxler et al., 2012). Thus, consistent with observations in other genetic models of partial complex I deficiency, a protective response to tissue injury may be observed (Oxler et al., 2012).

The Hq mouse exhibits an approximately 80% decrease in AIF content in all tissues. Defects are most profound in brain, with decreased complex I activity and complex I dependent respiration (Klein et al., 2002) and the development of disease in retina and brain (Hisatomi et al., 2001), reminiscent of human mitochondrial disease (Sevrioukova, 2011). In contrast, in heart, the metabolic defects are more subtle, although increased cardiac injury following *in vivo* ischemia-reperfusion (IR) and an increased susceptibility to heart failure in an aortic banding model of cardiac pressure overload have been reported (van Empel et al., 2005). The capacity to scavenge ROS is reported to be decreased in Hq mouse heart mitochondria compared to wild type (van Empel et al., 2005), suggesting that AIF has a potential antioxidant role. However, the net release of H_2_O_2_ is not altered in Hq mouse brain mitochondria compared to wild type (Chinta et al., 2009). This finding does not support an antioxidant role for AIF within mitochondria.

Cardiac mitochondria provide the energy to support heart function, whereas diseased and disabled mitochondria are a source of cardiomyocyte damage (Lesnefsky et al., 2001; Gustafsson and Gottlieb, 2008; Murphy and Steenbergen, 2008). IR results in damage to the electron transport chain that serves as a key source of ROS that exacerbate cardiac injury (Turrens, 2003; Chen et al., 2007). The net release of ROS from intact mitochondria represents a balance between ROS generation and the capacity of mitochondrial antioxidants (Turrens et al., 1991; Rigobello et al., 2006; Wenzel et al., 2008; Stanley et al., 2011). In the present study, we investigated if the net release of ROS from Hq mouse heart mitochondria is increased compared to wild type at baseline and if the genetic knockdown of AIF in Hq mice affects the net ROS generation and susceptibility to injury in the heart following the tissue stress of IR.

Translocation of AIF from mitochondria to the nucleus triggers caspase-independent cell death by inducing DNA damage (Yu et al., 2002; Sevrioukova, 2011; Natarajan and Becker, 2012). The mature form of AIF is anchored in the inner mitochondrial membrane (Ozaki et al., 2007; Chen et al., 2011). IR leads to a release of AIF from mitochondria into cytosol in isolated mouse heart, whereas administration of a calpain inhibitor prevents the loss of AIF from mitochondria (Chen et al., 2011). These results support that activation of mitochondrial localized μ-calpain is required to detach the AIF from the inner membrane (Ozaki et al., 2007; Chen et al., 2011) and indicate that retention of AIF within mitochondria provides cardioprotection during IR (Chen et al., 2011). The protection through retention of AIF within mitochondria can be due to a potential antioxidant role of the AIF or the prevention of AIF translocation to the nucleus. In the present study, mitochondria and nucleus were isolated from buffer perfused hearts to evaluate if IR increases the AIF translocation from mitochondria to nucleus. In the *in vivo* IR model, the length of the ischemic period was limited in that a relative long ischemic period could trigger a fatal arrhythmia occurrence in Hq mice (van Empel et al., 2005). Thus, a buffer perfused heart model was selected in the present study to avoid this confounding issue and to allow an ischemic period resulting in a moderate extent of mitochondrial and cardiac damage, relevant to *in situ* IR. Hq mice provide an experimental model to test the potential contribution of AIF to local mitochondrial protection compared to the deleterious cellular effects of nuclear translocation during IR.

## Methods

The experimental procedures conformed to the Guide for the Care and Use of Laboratory Animals and were approved by the Institutional Animal Care and Use Committees of Virginia Commonwealth University (VCU) and the McGuire Department of Veterans Affairs Medical Center.

### Preparation of mouse heart for perfusion

Wild type or Hq mice [2–3 months of age (22–28 g), male] were anesthetized with pentobarbital sodium (100 mg/g i.p.) and anti-coagulated with heparin (1 IU/g i.p.) (Chen et al., 2011). In this study, only male mice were used because Hq mice had a gender-dependent response in an experimental stroke model (Yuan et al., 2009). Compared to male Hq mice, female Hq mice exhibited markedly decreased brain injury after experimental stroke (Yuan et al., 2009). Since the AIF gene is located on the X chromosome, using male mice will avoid gender-dependent gene dosage effects on cardioprotection. Hearts were excised and retrograde perfused via the aorta in the Langendorff mode (constant pressure, 75 mmHg) with modified Krebs-Henseleit buffer (composition, in mM: 115 NaCl, 4.0 KCl, 2.0 CaCl_2_, 25 NaHCO_3_, 1.1 MgSO_4_· H_2_O, 0.9 KH_2_PO_4_, and 5.5 glucose) oxygenated with 95% O_2_+5% CO_2_. Cardiac function was monitored with a balloon inserted into the left ventricle using Powerlab (AD Instruments, Colorado Springs, CO). Heart rate was maintained at 420 bpm with pacing during the equilibration period. Pacing was stopped during global ischemia and restored at 15 min reperfusion. In the IR group, hearts were buffer-perfused for 15 min, followed by 30 min global ischemia at 37°C within 30 min reperfusion (mitochondrial isolation) or 1 h reperfusion (infarction measurement). In the time control group, hearts were buffer-perfused without IR. Myocardial infarct size was determined using TTC staining (Chen et al., 2006). Coronary effluent was collected to determine LDH activity in each group (Chen et al., 2006).

### Isolation of cardiac mitochondria from the mouse heart with protease

Trypsin was used in the mitochondrial isolation protocol in order to remove potential contamination from AIF located in the cytosol (Chen et al., 2011). The mouse heart was harvested and immediately placed in cold buffer A [100 mM KCl, 50 mM 3-(N-morpholino) propanesulfonic acid (MOPS), 1 mM EGTA, 5 mM MgSO_4_, 1 mM ATP]. Cardiac tissue was homogenized with a polytron at 10,000 rpm and incubated with trypsin (5 mg/g) for 15 min. Cold buffer B (0.2% BSA + buffer A) was then added into the homogenate. The homogenate was centrifuged at 500 × g for 10 min. The supernatant was centrifuged at 3000 × g for 10 min. to pellet mitochondria. The mitochondrial pellet was washed and suspended in 100 mM KCl, 50 mM MOPS, and 0.5 mM EGTA.

### Isolation of nucleus from buffer-perfused mouse hearts

The polytron pellet was used to isolate nuclear components using a commercial kit from Thermo Scientific (Pittsburgh, PA, catalog # 78835) using supplied solutions and differential centrifugation according to instructions provided.

### Measurement of oxidative phosphorylation and enzyme activities in isolated mitochondria

Oxygen consumption by mitochondria was measured using a Clark-type oxygen electrode at 30°C using glutamate + malate (complex I substrate) or succinate + rotenone (complex II substrate) as donors (Lesnefsky et al., 1997). Respiratory enzyme activities [NADH-decylubiquinol oxidoreductase, rotenone sensitive (complex I)]; NADH ferricyanide oxioreductase (NFR, flavoprotein portion of complex I); Succinate-decylubiquinone oxidoreductase (complex II); and citrate synthase were measured in detergent solubilized mitochondria according to the method of Dr. Hoppel as previously described (Krahenbuhl et al., 1991; Lesnefsky et al., 1997; Chen et al., 2008).

### Detection of H_2_O_2_ production from isolated mouse heart mitochondria

H_2_O_2_ production by isolated mitochondria was measured using the oxidation of the fluorogenic indicator Amplex red in the presence of horseradish peroxidase without exogenous SOD (Chen et al., 2003). Glutamate + malate and succinate + rotenone were used as complex I and complex II substrates, respectively. Rotenone and antimycin A were used to detect the maximal H_2_O_2_ generation from complex I and complex III, respectively.

### Determination of mitochondrial calcium retention capacity

Mitochondrial calcium retention capacity (CRC) was used to reflect opening of the mitochondrial permeability transition pore (MPTP) in isolated mitochondria (Chen et al., 2012a). CRC was studied in the single cell fluorimeter (PerkinElmer, Waltham, Massachusetts) using repetitive calcium pulses (Chen et al., 2012a). Freshly isolated mitochondria (0.25 mg) were incubated in buffer (150 mM sucrose, 50 mM KCl, 2 mM KPi, and 20 mM Tris/HCl, pH 7.4) for 90 s with stirring at 30°C with 0.5 μM calcium green. Succinate (5 mM) was used as substrate. Pulses of calcium (5 nmoles) were added at 60 s intervals. The number of pulses that resulted in calcium release indicated the onset of MPTP.

### Western blot analysis

Particle free cytosol, purified mitochondria, and nucleus were boiled for 5 min. in buffer including 4% (w/v) SDS, 1 mM 2-mercaptoethanol, 10 mM Tris/HCl (pH 6.8) and 10% (w/v) glycerol. Equal amounts of protein were loaded onto 4–15% or 4–20% SDS-PAGE (dependent on molecular weight of proteins), electrophoresed and transferred to a PVDF membrane. The membranes were first blocked by 5% non-fat milk for 1 h followed by exposure to primary antibodies overnight (Chen et al., 2011). Antibodies to AIF, PARP-1, GAPDH, lamin, and subunit IV of cytochrome oxidase were purchased from Cell Signaling Technology (Danvers, MA). Monoclonal PAR antibody was purchased from Millipore (Billerica, MA). The blots were incubated with peroxidase conjugated anti-rabbit or anti-mouse secondary antibody for 1 h prior to ECL detection (GE Healthcare Life Science, Pittsburgh, PA). The intensity of blotting was quantified by Fuji Film Image station (Edison, NJ).

### Statistical analysis

Data were expressed as the mean ± standard error of the mean. Differences among four groups were compared by one-way analysis of variance with *post-hoc* comparisons performed using the Student-Newman-Keuls test of multiple comparisons (*Sigmastat 3.5*, ProgramPaketet, Gothenburg, Sweden). Differences in CRC and H_2_O_2_ between wild type and Hq mice were compared by unpaired student *t*-test. A difference of *p* < 0.05 was considered significant.

## Results

### IR decreased oxidative phosphorylation in both wild type and Hq heart mitochondria

There were no differences in the rate of oxidative phosphorylation between time control wild type and Hq mitochondria when glutamate + malate (complex I) or succinate + rotenone (complex II) were used as substrates. IR decreased the ADP-stimulated respiration in mitochondria from both wild type and Hq using either substrate (Table 1). The rate of dinitrophenol (DNP) uncoupled respiration was also decreased in both wild type and Hq mice following IR (Table 1), supporting that IR damages the electron transport chain. Interestingly, the rate of oxidative phosphorylation with glutamate + malate in Hq hearts was decreased following IR compared to corresponding wild type (Table 1), whereas the rate of succinate oxidation was similar in Hq and wild type hearts following IR (Table 1). These results indicated that IR led to additional decreases in respiration in Hq mouse heart mitochondria when NADH-dependent substrates were used.

**Table 1:** **The rate of oxidative phosphorylation in wild type and Hq mitochondria following IR**.

**Mice**	***n***	**State 3**	**State 4**	**RCR**	**ADP/O**	**DNP**
**GLUTAMATE + MALATE WAS USED AS COMPLEX I SUBSTRATE (nAO/min/mg PROTEIN)**						
WT-TC	*N* = 8	324 ± 11	56 ± 5	6.2 ± 0.8	2.73 ± 0.108	341 ± 18
WT-IR	*N* = 8	271 ± 10^*^	56 ± 4	5.1 ± 0.5	2.80 ± 0.09	281 ± 11^*^
Hq-TC	*N* = 6	315 ± 27	51 ± 9	6.7 ± 0.8	2.80 ± 0.08	321 ± 40
Hq-IR	*N* = 7	224 ± 12^*^^†^	52 ± 9	5.3 ± 1.1	2.76 ± 0.13	229 ± 16^*^^†^
**SUCCINATE + ROTENONE WAS USED AS COMPLEX II SUBSTRATE (nAO/min/mg PROTEIN)**						
WT-TC	*N* = 8	474 ± 14	144 ± 3	3.3 ± 0.1	2.00 ± 0.07	435 ± 12
WT-IR	*N* = 8	266 ± 11^*^	133 ± 6	2.0 ± 0.1^*^	1.84 ± 0.05	339 ± 14^*^
Hq-TC	*N* = 6	496 ± 20	133 ± 18	3.4 ± 0.2	2.08 ± 0.06	471 ± 30
Hq-IR	*N* = 7	268 ± 15^*^	124 ± 6	2.2 ± 0.1^*^	2.07 ± 0.09	346 ± 25^*^

*Mean ± s.e.m*.

* p < 0.05 vs. corresponding time control (TC);

† *p < 0.05 vs. WT-IR (ischemia-reperfusion). RCR, respiratory control ratio. DNP (0.3 mM), dinitrophenol to measure the rate of uncoupled respiration*.

### IR decreased complex I activity in wild type but not Hq mouse heart mitochondria

In order to test if IR led to further damage to complex I; NADH:decylubiquinol oxidoredutase, NFR, and complex II activities were measured. Complex I activity [shown as the ratio of complex I/CS (citrate synthase), Table 2] was decreased in wild type following IR compared to time control. However, complex I activity was not decreased in Hq mitochondria following IR compared to its corresponding time control. The NADH dehydrogenase activity (NFR) was not decreased in either wild type or Hq mice following IR (Table 2), consistent with previous study (Chen et al., 2007; Szczepanek et al., 2011). NADH dehydrogenase activity was slightly higher in Hq mouse heart mitochondria following IR compared to corresponding wild type (Table 2). The physiological significance of this subtle difference is unclear. IR did not alter complex II activity in wild type or Hq mice (Table 2). These results support that IR leads to a complex I defect in wild type mouse heart mitochondria.

**Table 2:** **The enzyme activities of Complex I and II in wild type and Hq mitochondria with and without IR**.

**Mice**	***n***	**CS**	**Complex I/CS**	**NFR/CS**	**CII/CS**	**CII+Q/CS**
WT-TC	*N* = 8	2934 ± 1.4	0.200 ± 0.019	0.656 ± 0.055	0.039 ± 0.011	0.111 ± 0.020
WT-IR	*N* = 8	2937 ± 1.9	0.142 ± 0.010^*^	0.542 ± 0.029	0.059 ± 0.008	0.142 ± 0.012
Hq-TC	*N* = 6	2751 ± 1.7	0.195 ± 0.019	0.767 ± 0.050	0.045 ± 0.010	0.132 ± 0.026
Hq-IR	*N* = 7	2545 ± 1.7	0.174 ± 0.019	0.793 ± 0.081^†^	0.039 ± 0.010	0.103 ± 0.027

*Mean ± s.e.m*.

* p < 0.05 vs. WT-TC;

† *p < 0.05 vs. WT-IR. NFR, NADH dehydrogenase; CS, citrate synthase. Complex II activity (CII) was determined in the presence and absence of exogenous decylubiquinone (Q)*.

### The generation of H_2_O_2_ was decreased in Hq mouse heart mitochondria following IR

**Figure 1:**
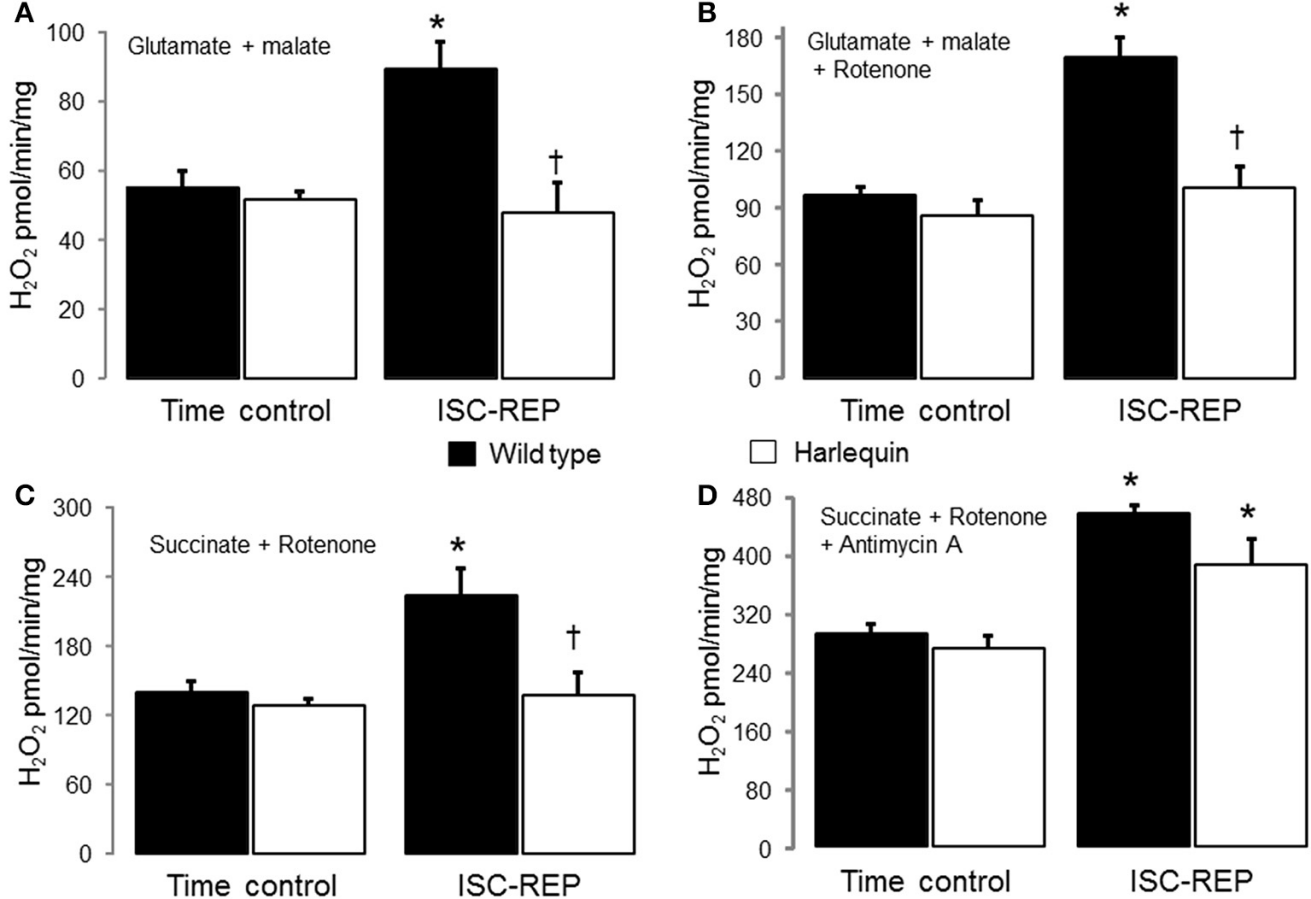
**Ischemia-reperfusion (IR) increases the net H_2_O_2_ production in wild type but not in Hq mouse cardiac mitochondria**. There were no differences in the net release of H_2_O_2_ between wild type and Harlequin mouse heart mitochondria from non-ischemic hearts. IR increased the generation of H_2_O_2_ in wild type but not in Harlequin mice compared to time control **(A)**. The maximal H_2_O_2_ generation from complex I was measured using rotenone to inhibit complex I. An AIF deficiency in Harlequin mice did not alter the maximal H_2_O_2_ generation from complex I compared to wild type in control heart **(B)**. The maximal H_2_O_2_ generation from complex I was also decreased in Harlequin mice following IR compared to wild type **(B)**. Knock down of AIF in Harlequin mice did not alter the H_2_O_2_ generation using succinate + rotenone as complex II substrates compared to wild type control. The H_2_O_2_ generation was decreased in Harlequin mice following IR compared to wild type **(C)**. The maximal H_2_O_2_ generation from complex III in the presence of antimycin A was not decreased in Harlequin mice following IR compared to wild type **(D)**. Data are expressed as mean ± s.e.m.; ^*^p < 0.05 vs. time control; ^†^*p* < 0.05 vs. wild type IR.

There were no differences in H_2_O_2_ generation between time control wild type and Hq heart mitochondria using complex I (Figure 1A) or complex II substrates (Figure 1C). IR markedly increased the production of H_2_O_2_ in wild type but not in Hq mouse heart mitochondria with either a complex I or complex II substrate (Figures 1A,C). Compared to time control, inhibition of complex I using rotenone dramatically increased H_2_O_2_ generation in wild type mouse heart following IR (Figure 1B). In contrast, rotenone inhibition did not increase the H_2_O_2_ generation in Hq mouse heart following IR (Figure 1B). The maximal ROS generation from mitochondria was induced with antimycin A inhibition. Inhibition of complex III using antimycin A increased the H_2_O_2_ generation in both wild type and Hq mouse heart following IR vs. time control (Figure 1D). However, there were no differences in ROS generation between wild type and Hq mice with or without IR.

### Cardiac injury was decreased in Hq mouse heart following IR

**Figure 2:**
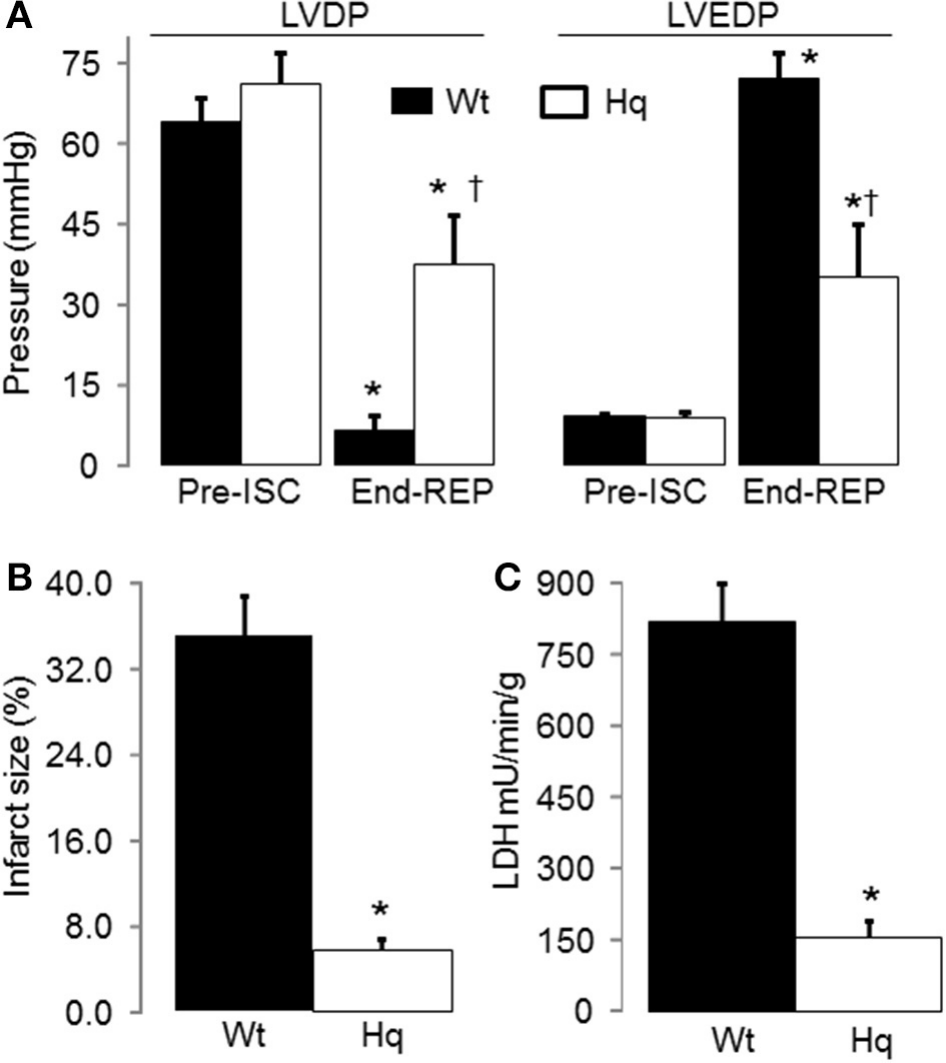
**IR led to decreased cardiac injury in Harlequin mice compared to wild type**. Heart rate was maintained at 420 bpm by pacing during 15 min equilibration perfusion. The pacing was stopped during ischemia and resumed at 15 min reperfusion. The recovery of myocardial contractile function during reperfusion, shown by the improvement in left ventricular developed pressure (LVDP mmHg, **A**), was improved in Harlequin mice compared to wild type. The diastolic function was also improved in Harlequin mice following IR, reflected by a decrease in left ventricular end-diastolic pressure (LVEDP) at the end of reperfusion **(B)**. The cardiac injury during IR was decreased in Harlequin mice as shown by a smaller infarct size **(C)** and less LDH release into coronary effluent. Data are expressed as mean ± s.e.m.; ^*^p < 0.05 vs. time control (TC); ^†^*p* < 0.05 vs. wild type untreated IR.

**Figure 3:**
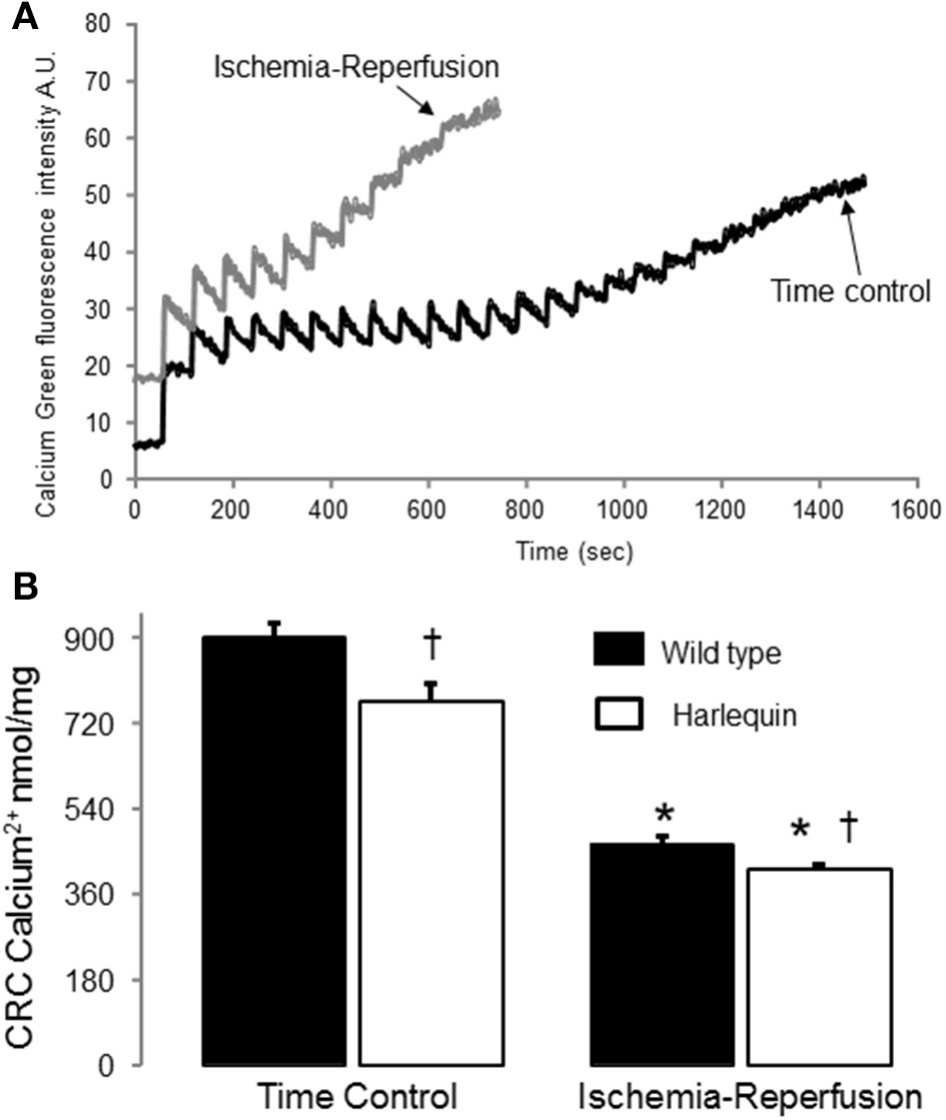
**IR decreased the CRC in wild type and Harlequin cardiac mitochondria**. Original tracings of CRC measurement in wild type heart mitochondria were shown in panel **(A)**. The CRC was decreased in control Harlequin mouse heart mitochondria compared to wild type **(B)**, suggesting that the sensitivity to MPTP opening was increased in Harlequin mice in the basal condition. IR led to decreased CRC in both wild type and Harlequin mice compared to time control **(B)**. Compared to wild type, IR led to a slight further decrease in CRC in Harlequin mice **(B)**. Data are expressed as mean ± s.e.m.; ^*^p < 0.05 vs. time control; ^†^*p* < 0.05 vs. wild type IR.

In the buffer perfused hearts, myocardial injury was decreased in the Hq mouse heart following IR compared to wild type. Knock down of AIF content in Hq mouse heart did not affect the cardiac function before ischemia (Figure 2A). IR decreased left ventricular developed pressure (LVDP) in both wild type and Hq hearts vs. time control. Systolic function was improved in Hq hearts vs. wild type during reperfusion (Figure 3A). Strikingly, the infarct size was also much smaller in Hq mice than in wild type (Figure 2B). The release of LDH into coronary effluent was much lower in Hq mice than in wild type (Figure 2C), also indicating less necrosis.

### IR decreased the CRC in both wild type and Hq mouse heart mitochondria

The CRC (Figure 3A) was decreased in mitochondria from non-ischemic Hq mice compared to wild type (Figure 3B), suggesting that a decrease in AIF content within mitochondria sensitizes to calcium-stimulated MPTP opening. Although there was a slight difference in the CRC between Hq and wild type following IR (Figures 3A,B), the small magnitude of this difference may not exert a significant impact on cardiac injury during IR.

### IR activated PARP-1 in wild type but not in Hq mice

**Figure 4:**
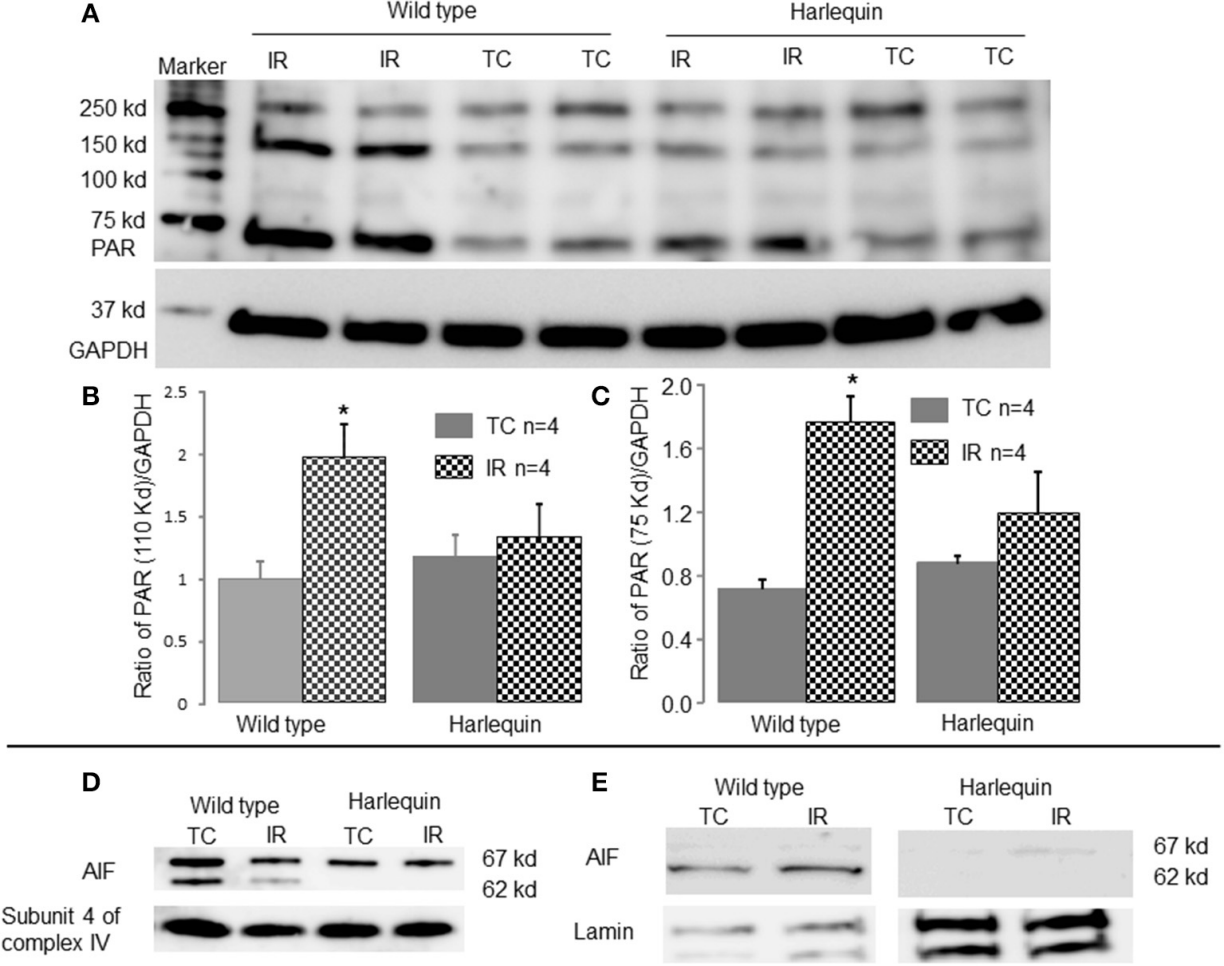
**IR increases AIF translocation from mitochondria to nucleus in wild type**. The content of poly (ADP-ribose) (PAR) was markedly increased in wild type mice following IR compared to time control, indicating that IR activated PARP-1 **(A–C)**. There were no differences in the PAR content in Hq mice between time control and mice following IR **(A–C)**. GAPDH was used as a cytosol marker for loading control. The precursor form of AIF (67 kd) and the mature form of AIF (62 kd) were detected in wild type mouse heart mitochondria **(D)**. Compared to control, IR markedly decreased the content of the mature form of AIF (62 kd band) in wild type mice **(D)**. The mature AIF content in Harlequin mice was of course decreased compared to wild type **(D)**. In wild type, IR increased AIF content (62 kd) in nucleus compared to time control, indicating a translocation of AIF from mitochondria to nucleus **(E)**. Subunit 4 of cytochrome oxidase and lamin were used as protein loading control for mitochondria and nucleus, respectively.

Activation of PARP-1 during IR increases the generation of PAR [Poly (ADP-ribose) (PAR)] that is transferred to cytosol and mitochondria (Sevrioukova, 2011). The content of PAR was markedly increased in wild type following IR compared to time control (Figures 4A–C), indicating that IR leads to PARP-1 activation. In contrast, IR did not alter the PAR content in Hq mice following IR (Figures 4A–C).

### IR decreased the AIF content within mitochondria and increased nuclear AIF content in wild type mice

The precursor of AIF (67 kd) is nuclear-encoded and subsequently transported into the mitochondrial matrix via its mitochondrial targeting sequence (Sevrioukova, 2011). The mature form of AIF (62 kd) is formed in the matrix through cleavage of precursor protein via a mitochondrial matrix peptidase (Sevrioukova, 2011). The mature AIF (62 kd) is transferred into the mitochondrial intermembrane space through the Tim23 protein (Sevrioukova, 2011). Consistent with these concepts, two AIF bands are detected in non-ischemic wild type mouse heart mitochondria (Figure 4D). In contrast, the mature form of AIF (62 kd) is almost undetectable in Hq mouse heart mitochondria (Figure 4D), confirming the lower content of AIF within mitochondria. The content of AIF within mitochondria was decreased in wild type mice following IR compared to time control (Figure 4D). The AIF content (62 kd) in nucleus was increased in wild type hearts following IR (Figure 4E).

## Discussion

In the present study, a deficiency of AIF within Hq mouse heart mitochondria does not increase the net release of H_2_O_2_ compared to wild type, consistent with reports in brain mitochondria. IR leads to increased net release of ROS from wild type heart mitochondria compared to non-ischemic controls. In contrast, the net release of ROS from Hq heart mitochondria is unchanged following IR. Thus, genetic knockdown of AIF within mitochondria does not increase the net release of ROS from the electron transport chain. These findings suggest that AIF less likely functions as a key mitochondrial antioxidant in the heart, especially following the stress of IR. The PARP-1 is activated in wild type but not in Hq mice following IR. Translocation of the AIF from mitochondria to the nucleus is increased in wild type but not in Hq mice following IR. The decrease in cardiac injury in Hq mouse heart accompanied by less AIF translocation to the nucleus suggests that the amount of AIF that relocates to the nucleus, rather than the AIF content within mitochondria, is the key factor that contributes to cardiac injury during IR.

### Complex I damage during IR

Complex I activity is decreased in heart mitochondria following both *in vivo* (Rouslin and Millard, 1980; Rouslin, 1983) and *in vitro* IR (Lesnefsky et al., 2001; Gustafsson and Gottlieb, 2008; Murphy and Steenbergen, 2008). In the present study, IR leads to decreased complex I activity without alteration in NADH dehydrogenase activity (NFR). These results indicate that ischemia likely damages complex I at the iron sulfur centers (Chen et al., 2008) distal to the flavoprotein, in line with previous studies (Ohnishi and Trumpower, 1980; Chen et al., 2006; Zhou et al., 2006; Szczepanek et al., 2011).

**Figure 5:**
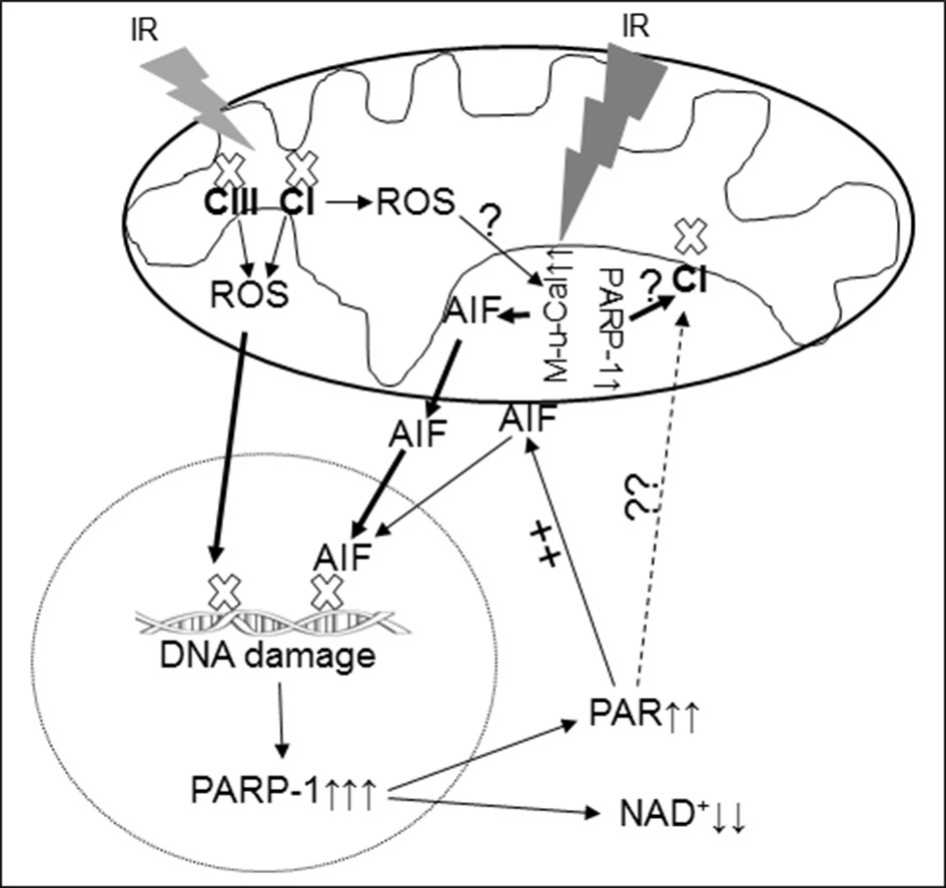
**Depiction of the PARP-1 activation and AIF translocation during IR**. IR damages the electron transport chain and increases the ROS generation from complex I and complex III and activates mitochondrial μ-calpain (M-μ-Cal) to induce release of AIF from the inner mitochondrial membrane. Translocation of AIF from mitochondria to nucleus enhances DNA damage and PARP-1 activation, in turn further reducing NAD^+^ content. Oxidative stress during IR leads to DNA damage DNA that activates the PARP-1 that leads to decreased NAD^+^ content in cytosol and increased generation of PAR within nucleus. The PAR is released into cytosol and relocates to mitochondria and facilitates AIF release from the outer mitochondrial membrane. The activated PARP-1 may contribute to complex I defect through an indirect mechanism. IR may activate mitochondrial localized PARP-1 to induce direct complex I damage in cardiac mitochondria. CI, complex I; CIII, complex III; M-μ-Cal, mitochondrial μ-calpain.

Oxidative modification of complex I by nitrosation (Burwell et al., 2006) or glutathionylation (Hurd et al., 2008) or the modification of its inner membrane environment via depletion of cardiolipin (Paradies et al., 2004) all contribute to decreases in activity. Mitochondrial AIF content also affects complex I activity, especially in brain and retina (Klein et al., 2002; van Empel et al., 2005). Depletion of AIF also decreases complex I activity in heart mitochondria (Pospisilik et al., 2007). However, the effect of lower expression of AIF in Hq mice on heart mitochondrial complex I activity is not consistent (Szczepanek et al., 2013). Thus, an AIF deficiency may affect complex I activity in a tissue-dependent manner. Genetic depletion of PARP-1 protects complex I activity in mouse heart following IR (Zhou et al., 2006), indicating that PARP-1 activation contributes to the complex I defect during IR. In the present study, IR decreases complex I activity accompanied by an activated PARP-1 in wild type mice. In contrast, IR does not decrease complex I activity in Hq mice. PARP-1 is also not activated in Hq mice following IR. These results support that activation of PARP-1 contributes to the complex I defect during IR. Since PARP-1 is considered as a nuclear protein, complex I inhibition by PARP-1 activation appears to present a challenge (Figure 5) (Zhou et al., 2006). Recently, a mitochondrial localized PARP-1 has been identified (Rossi et al., 2009). Thus, activation of PARP-1 may directly regulate complex I activity (Figure 5).

### Complex I inhibition and cardioprotection during IR

The ischemia-damaged respiratory chain including complex I, is a key source of ROS that increases cardiac injury (Turrens, 2003; Chen et al., 2007). Blockade of proximal electron transport using amobarbital before ischemia protects complex I and decreases cardiac injury during reperfusion, supporting that preservation of complex I activity reduces cardiac injury (Chen et al., 2006). Transient partial (Xu et al., 2014) or complete (Stewart et al., 2009; Chen et al., 2012b) blockade of complex I at the onset of reperfusion decreases myocardial injury in buffer perfused hearts, indicating that a temporary complex I inhibition is also beneficial for cardiac recovery. However, persistent, severe complex I inhibition is detrimental to the heart both at baseline and for recovery during reperfusion (Karamanlidis et al., 2013). In contrast to wild type, the complex I activity is not altered in Hq mice following IR. The lack of damage to complex I during IR in Hq mice may lead to decreased ROS generation that contributes to the observed decrease in cardiac injury in Hq mice. Alteration of mitochondrial antioxidants including thioredoxin reductase-2 significantly affects a release of H_2_O_2_ from mitochondria. The IR-induced complex I damage may also increase H_2_O_2_ by inhibiting thioredoxin reductase-2 through alteration of its redox state (Rigobello et al., 2006; Horstkotte et al., 2011; Stanley et al., 2011). The increased oxidative stress will favor activated μ-calpain to cleave AIF and facilitate its release from mitochondria (Norberg et al., 2010).

### Translocation of AIF from mitochondria to nucleus increases cardiac injury during IR

The mature form of AIF (62 kd) is anchored at the inner mitochondrial membrane within the intermembrane space (Otera et al., 2005). Release of AIF from the mitochondria and translocation to the nucleus to activate caspase-independent cell death is a multistep process. First, the mature AIF bound within mitochondria on the inner membrane requires liberation. Cleavage of the mature 62 kd form of AIF by activated mitochondrial calpains (Ozaki et al., 2007), *t*-bid (Cabon et al., 2012) or other proteases can liberate AIF from the inner membrane, with release of a truncated, approximately 57 kd AIF peptide. Next, permeation of the outer mitochondrial membrane is required for AIF release (Ozaki et al., 2007). IR increases MPTP opening as a mechanism of increased outer membrane permeability (Weiss et al., 2003), with oxidative stress is a key contributor to the increased susceptibility to MPTP opening during IR (Weiss et al., 2003; Halestrap et al., 2004; Chen et al., 2012a). In contrast, activation of PARP-1 is not involved in the permeation of the outer mitochondrial membrane during IR (Schriewer et al., 2013). The decreased AIF content in the purified mitochondria following IR supports that IR does lead to a loss of AIF from mitochondria. The increased MPTP opening in wild type mice during reperfusion favors a release of AIF from mitochondria into cytosol with subsequent translocation to the nucleus. It currently appears that even following MPTP, the released AIF is the 57 kd cleaved form, at least based upon calcium activation of mitochondrial calpains concomitant with MPTP. This area of calcium mediated injury deserves further consideration. As discussed above, IR activates PARP-1 in wild type but not Hq mice (Pacher and Szabo, 2007). Although activation of PARP-1 provides a beneficial effect to repair DNA damage, over activation of PARP-1 has a detrimental effect via consumption of NAD^+^ (Pacher and Szabo, 2007). PAR, which is generated by activation of PARP-1 within nucleus, is released into cytosol and subsequently relocates to mitochondria to induce AIF release from mitochondria (Pacher and Szabo, 2007). Interestingly, a portion of the mature AIF is also reported to be loosely attached on the mitochondrial outer membrane (Yu et al., 2006, 2009). The PAR can detach the AIF from the outer membrane (Figure 5) (Wang et al., 2009). Outer membrane bound-AIF has been identified in mouse heart mitochondria (Chen and Lesnefsky, unpublished data). Thus, activation of PARP-1 may increase AIF translocation to the nucleus through detachment of mature AIF from the outer membrane, in addition to release of the pool from the inner membrane via cleavage. The accumulation of AIF in the nucleus accompanied by increased cardiac injury in wild type mice following IR supports the proposal that the translocation of AIF from the mitochondria to the nucleus augments cardiac injury.

### Genetic inhibition of AIF expression in Hq mice decreases cardiac injury during IR

Although cardiac injury is increased in Hq mouse heart following *in vivo* IR compared to wild type controls (van Empel et al., 2005), cardiac injury is actually decreased in Hq mouse heart following *in vitro* IR. Several key differences likely contribute to these divergent results. In the present study, only male Hq mice were used based upon the rationale discussed in Methods, whereas both female and male Hq mice were included in the previous *in vivo* study (van Empel et al., 2005). This is important, since gender-related cardiac protection as well as gene dosage issues related to the X chromosome location of the *aif* gene can introduce variability. In the present study, only 2–3-month-old mice were used whereas middle-aged and elderly mice were used in the *in vivo* study (van Empel et al., 2005). *In vivo*, there is the additional impact of exogenous inflammatory cells (with or without AIF deficiency). Furthermore, *in vivo*, substrate utilization is uncontrolled. The metabolism of fatty acids *in vivo*, in contrast to glucose utilization in the current study, may have exacerbated the phenotype of mitochondrial defects present. Taken together, gender, age, exogenous cells and the different IR models likely resulted in the differences observed in our current study compared to the previous *in vivo* study. An isolated heart was used in order to focus on myocyte specific responses in the present study.

In summary, the key contribution of AIF to cardiac injury during IR is related to release from mitochondria and activation of programmed cell death via cytosolic transport, nuclear import and DNA cleavage. The findings in cardiac mitochondria from Hq mice compared to littermate controls support that a decreased content of AIF does not enhance ROS production from mitochondria nor augment cardiac injury at baseline nor during IR. Thus, AIF does not exert significant mitochondrial antioxidant protection during IR. The prevention of AIF translocation to nucleus is a potentially powerful approach to reduce cardiac injury.

### Conflict of interest statement

The authors declare that the research was conducted in the absence of any commercial or financial relationships that could be construed as a potential conflict of interest.
